# Editorial: Antibiotic allergy de-labelling and management

**DOI:** 10.3389/falgy.2023.1278690

**Published:** 2023-09-19

**Authors:** Gigia Roizen, Patrick E. Beeler, Miguel Park, Mariana Castells, Elina Jerschow

**Affiliations:** ^1^ Allergy and Immunology, Internal Medicine Department, Clínica Alemana de Santiago, Santiago, Chile; ^2^ Division of Occupational and Environmental Medicine, Epidemiology, Biostatistics and Prevention Institute, University of Zurich and University Hospital Zurich, Zurich, Switzerland; ^3^ Center for Primary and Community Care, University of Lucerne, Lucerne, Switzerland; ^4^ Division of Allergic Diseases, Mayo Clinic, Rochester, MN, United States; ^5^ Drug Hypersensitivity and Desensitization Center, Brigham and Women’s Hospital, Boston, MA, United States

**Keywords:** drug allergies, beta-lactam, de-labeling, graded challenges, antibiotics

**Editorial on the Research Topic** Antibiotic allergy de-labelling and management

Allergy to antibiotics is an important health care challenge that affects both the allergy sufferers and their immediate surroundings, but allergy labels are often unconfirmed. Antibiotics allergy-labeled patients may not be protected by the label since they are often unable to receive their first-choice treatment, opting for second line, more expensive alternatives, with increased complications. The community is also adversely affected by drug allergies labels, as second line antibiotics may lead to drug resistance in pathogenic bacteria. This Research Topic of Frontiers in Allergy focuses on the de-labelling and management of antibiotic allergies and the importance of testing patients who may no longer have such allergies.

There is a definite need for evaluations of patients reporting antibiotic allergy, as presented by Iuliano et al. Only 13% of the patients with reported allergy to beta-lactams tested positive either on skin test or through the graded challenge. This is slightly higher than the previously reported findings by Iammatteo et al. (1). It is possible that the higher percentage of patients in the study by Iuliano et al. with identified allergy was due to the methods used for the testing—skin test vs. direct challenges (1, 2). Skin testing tends to detect a higher number of allergic reactions to beta-lactams than the direct challenges, although some of them are possibly false-positive (1, 2). The findings on the safe administration of direct challenges in low-risk patients with beta-lactam allergies in a hospital setting were once again confirmed by Sunagawa et al.. In 96% of all patients, beta-lactam allergy label has been removed.

The review on the need for de-labeling of multiple antibiotic allergies by Li and Thong echoed the importance of testing patients with historical reactions to beta-lactams. The implementation of a penicillin de-labeling program across healthcare systems, with an approximately 95% success rate, has the potential to lead to substantial cost savings and promote more judicious antibiotic prescribing practices (Dong et al.).

In this context, achieving a highly specific diagnosis of drug allergies is of utmost importance to reduce unwarranted avoidance of entire classes of medications, particularly beta-lactams. The de-labeling of the patients with a reported beta-lactam allergy is also important when caring for special groups of patients: pregnant women or during peri-operative care (Ramsey).

Overall, the use of perioperative cephalosporins for most patients reporting an unverified penicillin allergy was safe, but demonstrated a higher risk for reactions (3%) in patients with a verified penicillin allergy (Ramsey).

Another often overlooked group were patients with hereditary angioedema (HAE). Wong et al. observed that 100% of HAE patients from Hong Kong were drug allergy free after testing.

The articles in the current Frontiers in Allergy Research Topic highlight not only the process of clarifying diagnoses but also emphasize the need of updating medical records to ensure that there is genuine change in clinical practice. The discrepancy between the electronic health record (EHR) allergy list and drug challenge test results contributes to inaccurate allergy labels, inappropriate drug-allergy alerts, and potentially ineffective or costly care (Lo et al.). To address this issue, natural language processing (NLP) was leveraged to automatically detect such discrepancies and provide real-time clinical recommendations through allergy reconciliation modules, as presented by Lo et al. in this Research Topic. They found that the combined use of NLP and clinical documentation effectively improved allergy documentation, leading to better treatment decisions.

The articles presented in this Research Topic shed light on the pressing need to advance antimicrobial stewardship through de-labeling patients with penicillin and other beta-lactams inaccurate allergy labels, as well as other drug allergies. Guidelines on risk stratification and the use of direct challenges for low-risk patients are described, which should provide a safe platform for their implementation by clinicians and all health care providers. Major challenges remain in the capture of antibiotic drug allergies in electronic medical records which are highlighted and the need for a new language which can standardize the presentations, diagnosis, management, and treatment recommendations. Collaborative efforts between healthcare institutions, allergists, immunologists, clinical pharmacologists, and multidisciplinary teams are pivotal in optimizing antibiotic prescribing practices, improving patient outcomes, and decreasing antimicrobial resistance.
